# The Cost of War on Public Health: An Exploratory Method for Understanding the Impact of Conflict on Public Health in Sri Lanka

**DOI:** 10.1371/journal.pone.0166674

**Published:** 2017-01-12

**Authors:** Sandy A. Johnson

**Affiliations:** Josef Korbel School of International Studies, University of Denver, Denver, Colorado, United States of America; University of Colorado, UNITED STATES

## Abstract

**Purpose:**

The direct impact of protracted conflict on population health and development is well understood. However, the extent of a war's impact on long-term health, and the opportunity costs, are less well understood. This research sought to overcome this gap by asking whether or not health outcomes in Sri Lanka would have been better in the absence of a 26-year war than they were in the presence of war.

**Methods:**

A counterfactual model of national and district-level health outcomes was created for Sri Lanka for the period 1982 to 2002. At the national level, the model examined life expectancy, infant mortality rate (IMR), and maternal mortality ratios (MMR). At the district level, it looked at IMR and MMR. The model compared outcomes generated by the counterfactual model to actual obtained health outcomes. It looked at the rate of change and absolute values.

**Results:**

The analysis demonstrated that war altered both rate of change and absolute health outcomes for the worse. The impact was most clearly evident at the district level. IMR was poorer than predicted in 10 districts; of these 8 were outside of the conflict zone. The MMR was worse than expected in 11 districts of which 9 were not in the conflict zone. Additionally, the rate of improvement in IMR slowed as a result of war in 16 districts whereas the rate of improvement in MMR slowed in 9.

**Conclusion:**

This project showed that protracted conflict degraded the trajectory of public health in Sri Lanka and hurt population health outside of the conflict zone. It further provided a novel methodology with which to better understand the indirect impact of conflict on population health by comparing what is to what could have been achieved in the absence of war. In so doing, this research responded to two public health challenges by providing a tool through which to better understand the human and opportunity costs of war and by answering a call for new methodologies.

## Introduction

Although approximately 55,000 people are killed directly in violent conflict each year, this number represents only a portion of the total deaths associated with war [1]. Morbidity and mortality is not limited to the battlefield or those directly killed by combatants [2–8]. Mortality and morbidity that stem from the indirect effects of war range from being equal to the number of deaths experienced in war, to being over nine times greater than the death rate due to violence [2, 7–9]. Such deaths occur in combatant and civilian populations alike as crude death rates, maternal mortality rates, and infant mortality increase during war and in the post-conflict period rise [2, 6, 10–12]. The destruction caused by war creates prolonged instability, and brings high direct- and opportunity-costs that impact household and national development [3, 5, 6, 8, 13–16]. In the best cases, a development trajectory slows but may recover; in the worst cases, intractable poverty and a conflict spiral result.

Although the mechanisms through which conflict can impact health and human development are understood, our ability to determine the magnitude of war’s impact on public health over time remains flawed. This is due to the varying nature of war, the complexity of the development process, and a paucity of methodology to account for direct and indirect costs of battle.

This paper presents a methodology that attempts to capture the direct and indirect impact of protracted conflict on public health during the war in Sri Lanka by creating a counterfactual model that compares what was achieved in health outcomes to what could have been achieved in the absence of war.

Conflict does not impact all states in the same way. National context and capabilities, and the nature of the conflict itself influence development progress and population health during and after the course of a war [17–19]. Several studies show that the worst losses to health occurred in areas that either directly experience war or bordered areas of high-intensity conflict [2, 8, 20, 21]. Others show more wide-spread impacts [3, 12]. In some cases, the combat leads to large and ubiquitous impacts across society [1, 2, 6–8, 20]. In other cases, large segments of society are left seemingly untouched by war and may see different aspects of development actually continue to improve [17, 22].

Sri Lanka appears to be an example of the latter in that multiple indicators of human development continued to improve at the national level throughout a protracted civil war that began in 1983 and ended in 2009. By 1982, Sri Lanka reached an average life expectancy at birth of 68 years, an infant mortality rate (IMR) of 34 per 1,000 live births, a maternal mortality ratio of 0.6 per 1,000 live births and a literacy rate of 87%—figures comparable to those obtained in middle and high income nations. It did this on a GDP per capita of $466 (constant 2000 US$)[23]. By war’s end, life expectancy was 72 years, and the national IMR had dropped to 16.9 deaths per 1,000 live births [23]. During the course of the war, Sri Lanka’s Human Development Index (HDI) improved from 0.649 to 0.752 [24]. The economy grew at an average of 3.7% per year during the conflict. Beginning 1997, the World Bank reclassified Sri Lanka as a lower middle income country.

Does this then mean that the conflict had little negative impact on the course of human development in this country?

Lessons learned from other conflicts suggests not. The impact of conflict cannot readily be isolated or contained; one need consider the visible and physical impacts as well as the less visible opportunity costs of conflict. Analyses of conflict areas show that war has both immediate and long-term impact on human progress across multiple sectors [2, 3]. Although direct effects may be more easily observed and isolated in geographic regions in which actual fighting occurs, the insidious nature of conflict is such that its impact trickles over boundaries be they district boundaries within a country or national boundaries. Observers in Sri Lanka noted the deterioration of health, education and livelihoods experience in the Northern and Eastern provinces of the country throughout the course the civil war [25, 26]. Has this deterioration spilled over into Sri Lanka’s broader development course vis-à-vis population health? This research seeks to answer that question by exploring the impact conflict had on human health, as one aspect of human development, in Sri Lanka.

Methodology commonly employed to understand the war’s impact on health falls broadly into four categories: 1) qualitative descriptions of destruction and suffering [27]; 2) examinations of health outcomes in the final years of war or in the immediate aftermath [11, 20]; 3) analysis of health outcomes based on single data points from a year before the war and at war’s end [12, 17]; and 4) analysis of trends in outcomes observed during the course of war [6, 21]. While these approaches all offer insight into the dynamics of war and health, each has its limitations. The qualitative examination captures the lived experience and visceral realities of war but does not provide broad geographical coverage or generalizability, nor do such observations adequately capture change over time. The second method looks at only a small window of time and does not account for pre-war conditions. The third method uses few data points–health indicators from a year or two prior to the war and data from a year after the end of war. It does not account for pre-war momentum in development pathways, nor does it well handle the dynamics of protracted conflict and the multitude of changes that occur over decades. The final method captures dynamic shifts in trends over time but accounts only for the context of a nation at war rather than comparing the periods of war and peace. All of these methods fail to account for a nation-state’s trajectory prior to war and therefore fail to account for the loss of lives that could have been saved and opportunities lost for human development. The limitations of such methods are well known and relate to the dearth of reliable data from war-torn areas. Murray and colleagues point out that improved data availability, counterfactual modeling, and use of household survey data offer promising routes to better understand the impact of conflict on public health [9]. They explicitly state that in order to understand the full cost of war, including opportunity costs, one must measure what health outcomes would be in the absences of war. This paper attempts to do just that by creating a counter-factual model. I created the model by using pre-war trends to of health outcomes at national and district levels in Sri Lanka and compared those to obtained outcomes. I also examine quantitative health outcomes and qualitative measures of morbidity and mortality at national and district levels in order to add depth to my analysis.

My objectives were three-fold: 1. To differentiate the impact of war on health outcomes at national and district levels; 2. To understand whether conflict slowed the pace of change in Sri Lanka’s health at diverse spatial levels, and; 3. To identify the spatial pattern of health outcome in order to understand whether conflict impacts only proximate people and health systems.

## Background

Over the past 60 years, Sri Lanka realized and sustained levels of human development typical of the developed world, and proved to be a success story in terms of fostering and sustaining a high level of human well-being despite a modest GDP per capita and low- to moderate- economic growth (Table 1). National-level development indicators achieved high levels prior to the onset of war, indicating strong development performance, and continued to improve during the conflict period. The sustained progress is believed to be due to strong political commitment to social welfare programs, democratization, early and continued rural development, near-universal education, investment in female education, and sustained commitment to family planning [28–31].

**Table 1:** Health and Development Indicators for Sri Lanka: 1945–2011.

	1945	1972	1982	1992	2002	2011
**GDP/Capital ($US constant 2000)**	n/a	332.20	471.90	615.50	883.50	1402.10
**Infant Mortality Rate (per 1,000 live births)**	140	45.6	31	18	11	10.5
**Life Expectancy at Birth**	43 (1946)	63.9	69	69.7	72.5	74.9
**Maternal Mortality Ratio (per 1,000 live births)**	16.5	1.3	0.6	0.4 (1991)	0.14	n/a

Sources: Bjorkman 1985; Ministry of Healthcare and Nutrition (multiple years); World Bank 2013.

Despite the achievements in human development, there were clear social cleavages between the Sinhalese and Tamil populations. In 1983, ethnic tensions erupted into violent conflict between the Government of Sri Lanka (GoSL) and a Tamil militant group the Liberation Tigers of Tamil Eelam (LTTE). The first phase of this conflict, Eelam War I, ended when a truce was implemented in 1987. This lasted three years. During this time, the GoSL fought a two-year insurgency against the Janatha Vimukthi Peramuna (JVP). Eelam War II continued the hostilities between the GoSL and LTTE beginning in 1990 and ending in 1994 when a new Sri Lankan government took office on a peace platform. Again peace negotiations commenced but, as before, collapsed leading to Eelam War III from 1995 to 2002. The truce ushered in by the 2002 February Ceasefire Agreement (CFA) was short-lived. A tsunami devastated coastal communities around Sri Lanka in December, 2004. Many of the communities affected by the tsunami also suffered from the war. The conflict erupted again in late 2005. By 2006, fighting between the government and LTTE forces escalated. The GoSL formally stepped away from the CFA in January, 2008. The final Eelam war ended in May, 2009 with the GoSL declaring victory.

**Fig 1:**
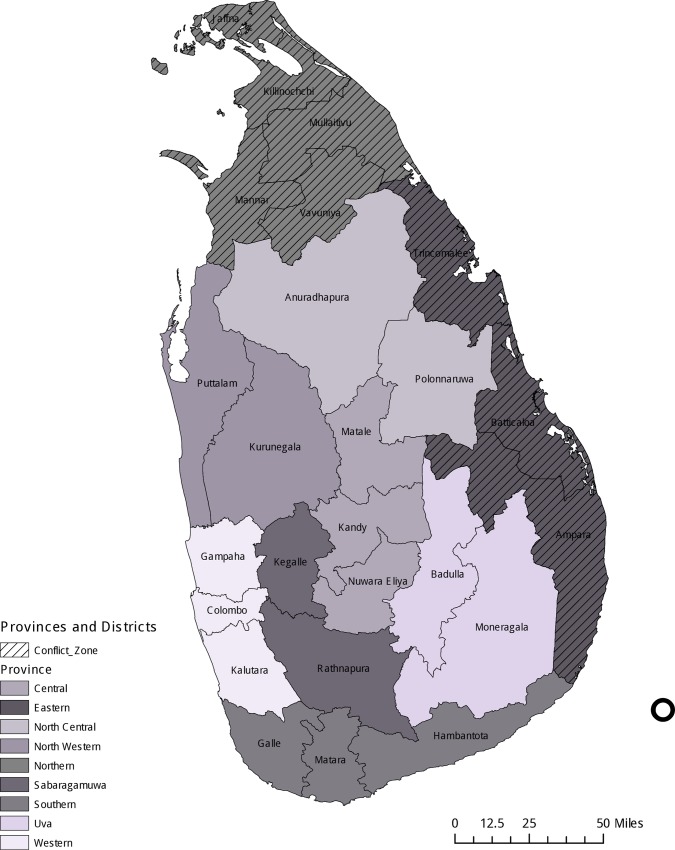
Sri Lanka Provinces and Districts.

The conflict was entrenched in the Northern and Eastern provinces, but battles and combatants spilled over into neighboring provinces and districts throughout the war (Fig 1). Control of land, lives, livelihoods and administrative policy was subject to the ever changing whims of war. This was particularly true in several districts within the Northern province that were collectively known as Vanni–the area made up of Mannar, Mullaitivu, Kilinochchi and Vavuniya districts. During the war, this area was captured and controlled by the LTTE. The Vanni served as both a fictive homeland and administrative center for the LTTE, and proved to have a comparatively stable boundary. Although the LTTE undertook state-building effort in theVanni, the GoSL continued to supply moderate health and educational resources to the embattled districts [30]. The remaining district in the Northern Province, Jaffna, is situated in a strategically important peninsula. The LTTE took control of Jaffna in 1987. The GoSL regained control of Jaffna in 1995. Development in Jaffna reignited to a limited degree following the GoSL regaining control in 1995. It was after the government regained control of Jaffna that LTTE shifted the epicenter of its activities to the Vanni.

The control of broad swaths of land within the Eastern Province changed hands multiple times throughout the course of the war although there was never a permanent LTTE homebase equivalent to Vanni. This vacillation between overlords resulted in an erratic approach to development that was dependent both upon the intensity of fighting, and which group controlled the territory at any given time. Development efforts undertaken by the GoSL in the east were largely concentrated in the urban areas and military garrisons in Trincomallee and Baticalloa. Although the Eastern Province was one of the main foci for the war, in comparison to the Northern Province, there were periods of comparative stability.

## Materials and Methods

This research was approved by the University of Denver Institutional Review Board Protocol Number 2009–1112. All data used are aggregated to the district level and contain no information at the individual level. The Ministry of Health and Nutrition gave permission to access data in accordance with the IRB protocol.

I obtained data on infant mortality rate per 1,000 live births (IMR) and maternal mortality ratios per 1,000 (MMR). National level measures were compiled from the Central Bank of Sri Lanka, Department of Census and Statistics, the Ministry of Health and Nutrition. I supplemented these with data from the United Nations Development Program and the World Bank. IMR and MMR at the third administrative tier, the district level, were compiled from data from the Central Bank of Sri Lanka, Department of Census and Statistics, and the Ministry of Healthcare and Nutrition. Data were available from 1975 forward. These data were gathered during field work in June and July 2009. In cases where the same year/data were available from different sources, I compared them to ensure consistency.

The reliability of the national level infant mortality data was difficult to assess for the post-1999 period. Domestic and international sources demonstrated remarkable disagreement. For example, World Bank data reported an IMR of 18.7 per 1,000 births in 2001 and 18.2 in 2002. The Annual Health Statistics published by the Ministry of Health and Nutrition (MoHN) reported a national rate of 12.2 per 1,000 births in 2001 (2002 not available). Because I was unable to ascertain whether or not the MoHN aggregation included data from the Northern and Eastern provinces–the provinces that experienced conflict—I used World Bank data for national IMR models and calculations.

The veracity of IMR data is unknown. Infant mortality is often under-reported especially in conflict areas because of weakened vital statistic registration and higher likelihood of non-hospital births [32, 33]. Although hospital births are common in much of Sri Lanka, this pattern was disrupted in the conflict areas because clinics were destroyed, fewer health professionals were available, and travel to facilities was increasingly difficult.

National data for the main causes of hospital morbidity and mortality came from the Annual Health Statistics report issued by the Ministry of Healthcare and Nutrition. Data in these reports are reported in five-year increments from 1990 to 2005. Because the 1990 data excluded information from the Northern and Eastern provinces, I limited my analysis to 1995 forward. Cause of morbidity and mortality are reported using collapsed International Classification of Disease (ICD) 9 codes and ICD10 codes. For example, the MoHN reports a category for traumatic injuries which includes ICD codes 800–904, 930–939, and 950–957. I could not obtain the disaggregated data.

I also obtained district-level data on causes of hospitalization and hospital-based death from the Ministry of Healthcare and Nutrition. As was true with the national data, data for districts in the Northern or Eastern provinces were only available from 1995 forward. My analysis of hospitalization and mortality focus on 1995 and 2002 due to the limitations described above.

In order to understand the impact conflict had on health, I adopted the methodology used by Sommers (2002) to explore the impact of conflict on education. First, I calculated average annual change in each indicator using data from five to ten years prior to 1983 depending upon data availability. I used this formula:

$$Average\;Rate\;of\;Change=\frac{\left(Rate^{present}–Rate^{past}\right)/\;Rate^{past}}{Number\;of\;Years}$$

Second, I created a counterfactual model projecting the average annual change from the baseline onto the 1982 outcome in order to determine the change one might expect had the conflict not occurred. I used the years 1999 and 2002 as conflict endpoints depending upon data availability.

I chose 2002 as the endpoint for my analysis for several reasons. First, district-level data were limited for the fourth phase of the war and its immediate aftermath. I was also concerned about the potential confounding in health indicators as a result of the 2004 tsunami. Eelam War III ended in 2002 and so represented one end-point of the conflict. Although the war re-ignited, I chose to limit my analysis to this period due to comparatively good quality and availability of the data. I report outcomes for 2002 and 1999, as the most complete data are available for that year. Third, I calculated the difference between the factual outcomes and the counterfactual models. Mullaitivu and Kilinochchi districts, both district in areas impacted by the war, were created in 1978 and 1984 respectively, and therefore do not have adequate data for the analysis described above. They are excluded from the counterfactual modeling.

Fourth, I supplement the quantitative findings by describing patterns in the primary causes of hospitalization and death at national and district levels. For clarity in reporting, I divided the districts into three categories: those that were the site of High Conflict where there were frequent military encounters, those of Intermittent Conflict which border the conflict zone, and areas of No Conflict.

Lastly, I created a conflict impact scale based on the district level analysis of IMR and MMR. Any district in which the actual rate of change in IMR/MMR was slower than that obtained in the counterfactual model was given a weight of one. Thus, a district that experienced a slower rate of change in IMR would receive a one; a district that experienced slower actual rate of change in both IMR and MMR would receive a two. I assigned a single point for each (IMR/MMR) if the actual outcome in 2002 was worse that the counterfactual model predicted. Thus, a district which experienced slower rates of change in both IMR and MMR, and in which the 2002 endpoint for IMR and MMR was worse than that predicted by the counterfactual model received a weight of 4, indicating high impact from conflict. I created a choropleth map to better visualize the spatial distribution of conflict impact. All maps in this research were created using ArcGIS ® 10.3. Boundary files came from the Global Administrative Areas database.

The counterfactual model assumes linear change based on the average annual change observed in the pre-war years. Even the best health datasets demonstrate that, over time, the changes IMR and MMR are filled with ‘noise’ and do not conform to a smooth, linear model. To account for these difficulties, I did two things. First, I examined a real world ‘counterfactual model’ to determine whether the Sri Lankans counterfactual predictions were realistic. I choose Costa Rica and Malaysia as examples of low income countries (in the early 1980s) which are noted for achievements in human development. I used World Bank Indicators for this analysis. Because sufficient MMR data were not available, I only looked at IMR. Sri Lanka’s counterfactual rate of change falls within the range exhibited by Costa Rica, a smaller nation with larger GDP/cap, and Malaysia, a larger nation that successfully staved off an ethnic conflict (S1 Table: Appendix 1).

I also performed a sensitivity analysis to determine whether the shape of the projected trend line impacted outcomes. In the sensitivity analysis, I used both exponential and natural log projections for counterfactual models and compared these to the linear projection. I found moderate consistency in MMR in that the linear, exponential and logarithmic projections agreed in all but seven of the provinces. In other words, whether or not the actual MMR was better or worse than the counterfactual was consistently predicted. The results were mixed for IMR. Of the 25 cases, less than half (11) showed consistent outcomes across all three models. At the national level, all models were consistent based on five-year trend in MMR but inconsistent for five-year IMR and all models using ten-year trend data (S2 Table: Appendix 2).

## Results

### IMR and MMR

#### National data

This war extracted a price on Sri Lanka. National level data suggest that the cost came in terms of overall national ability to reduce infant mortality and maternal mortality. Table 2 compares actual outcome for each health indicator to expected outcomes. The first column records the reported health outcome by year. The second and third columns show counterfactual outcomes based on averaging pre-war change for both five and ten years prior to conflict. The next three columns show the average annual change for five and ten years prior to the war, and the actual change during the course of the war. The final column is the interpretation.

**Table 2:** Actual and Predicted Health Outcomes, Sri Lanka 1982–2002.

Indicator	Actual Outcome	Counterfactual Outcomes		Trend			Interpretation			
		Based on 5-yr. trend	Based on 10-yr. trend	5 yr. pre-conflict average annual change	10-y.r pre-conflict average annual change	Ave. annual change during conflict				
1982 Infant Mortality Rate per 1,000 live births	34.00			-0.04	-0.04	-0.02	Improvements in IMR slowed during war. Absolute outcomes are worse than expected given pre-war trends.			
2000 Infant Mortality Rate per 1,000 live births	19.30	16.32	18.06							
2002 Infant Mortality Rate per 1,000 live births	18.20	15.04	16.83							
										
1982 Maternal Mortality Ratio per 1,000 live births	0.60			-0.08	-0.05	-0.04	Improvement in maternal mortality slowed during the war. Outcome is inconclusive.			
2002 Maternal Mortality Ratio per 1,000 live births	0.14	0.11	0.20							

Sources: Central Bank of Sri Lanka; Ministry of Health and Nutrition (multiple years); World Bank 2013

The IMR at the close of the third Eelam war, and the rate of change in IMR during the war, are both worse than expected given pre-war trends. The infant mortality rate was declining at an average of 3.5 per 100,000 between 1972 and 1982. This rate decreased to 2.3 per 100,000 during the conflict period. In other words, an additional 1.2 infants per 100,000 died every year due to the loss of momentum. The actual outcome shows a worse IMR than outcomes predicted by either the trend observed for five or ten years prior to the onset of the war.

National maternal mortality ratios decreased during the war. The 0.6 per 1,000 lives lost before the start of the war decreased to a remarkable 0.14 per 1,000 between 1982 and 2002. This outcome is better than expected given the average annual change observed between 1972 and 1982 in which the average annual decrease was 0.08 per 1,000. However, the rate of change for the conflict period, an average annual decrease of 0.4 per 1,000, was lower than the average rate of change in the five years prior to the war. Therefore, the achieved MMR of 0.14 per 1,000 in 1992 was worse than expected given the five-year counterfactual model.

#### District level data

Tables 3, 4, 5 and 6 provide a breakdown of IMR and MMR at the district level. Tables 3 and 5 note the level of each indicator prior to the onset of war and in 2002 (or 1999 depending upon data availability). The tables show the average annual change in each indicator based on data prior to war, and the average annual change in the indicator during the war years. The final columns show the value of each indicator in 2002 and the hypothetical value had prewar levels of change been maintained. Tables 4 and 6 provide a visual summary of the conflict induced outcomes. The ‘Rate of Change and IMR/MMR Worse than Predicted’ column indicates the most sever situation in which the actual health outcome and the rate of change are both worse that could have been obtained without war. Subsequent columns show whether both the rate of change and actual health outcome were better than predicted, or whether only the actual rate of change appeared worse than expected.

**Table 3:** Actual and Counterfactual IMR at the District Level, Sri Lanka 1982–2002.

		Actual IMR per 1,000 Live Births		Counterfactual Outcome based on Pre-war trend	Average Annual Change	
Conflict Class	District	1982	2002	2002	Pre-Conflict Average (1975 to 1982)	Average Change During Conflict
High Conflict Level	Batticaloa	27	15.2	6.2	-0.066	-0.019
	Jaffna	17	6	12.7	-0.027	-0.038
	Kilinochchi	n/a	3.9	n/a	n/a	n/a
	Mannar	27	6.9	13.1	-0.010	-0.030
	Mullaitivu	n/a	8.9	n/a	n/a	n/a
	Trincomalee	18	2.5	4.1	-0.060	-0.043
	Vavuniya	16	11.8	4.8	-0.058	-0.016
						
Intermittent Conflict	Ampara	20	7	3.5	-0.076	-0.031
	Anuradhapura	26	17.6	11.6	-0.034	-0.012
	Polonnaruwa	14	16.1	1.7	-0.086	0.031
	Puttalam	24	6.3	13.5	-0.032	-0.040
						
No Conflict	Badulla	34	15.9	10.7	-0.056	-0.025
	Colombo	50	16.2	n/a	n/a	
	Galle	34	10.9	13.3	-0.046	-0.032
	Gampaha	22	5.2	n/a	n/a	-0.037
	Hambantota	18	4.7	4.7	-0.065	-0.035
	Kaluthara	20	4	3.6	-0.082	-0.043
	Kandy	39	15.8	12.1	-0.065	-0.035
	Kegalle	29	9.2	7.4	-0.066	-0.035
	Kurunegala	29	10.8	14.4	-0.031	-0.032
	Matale	29	7.6	6.3	-0.058	-0.006
	Matara	33	5.9	18.7	-0.028	-0.042
	Moneragala	13	2	2.6	-0.076	-0.045
	Nuwara Eliya	49	16	16.1	-0.054	-0.036
	Ratnapura	43	13.5	17.6	-0.044	-0.033

Source: Central Bank of Sri Lanka; Dept. of Census and Statistics; Ministry of Healthcare and Nutrition (multiple years)

**Table 4:** Severity of Impact of Conflict at the District Level, Sri Lanka 1982–2002.

Conflict Class	District	Rate of change and IMR are worse than predicted	Rate of change and IMR are better than predicted	Rate of change is worse but IMR is better	Summary of Conflict Induced Outcome
		** **	** **	** **	** **
**High Conflict**	Batticaloa	X			Rate and IMR are worse
	Jaffna		X		Rate and IMR are better
	Kilinochchi				Unable to determine
	Mannar		X		Rate and IMR are better
	Mullaitivu				Unable to determine
	Trincomalee			X	Rate is worse; IMR is better
	Vavuniya	X			Rate and IMR are worse
					
**Intermitent Conflict**	Ampara	X			Rate and IMR are worse
	Anuradhapura	X			Rate and IMR are worse
	Polonnaruwa	X			Rate and IMR are worse
	Puttalam		X		Rate and IMR are better
					
**No Conflict**	Badulla	X			Rate and IMR are worse
	Colombo				Unable to determine
	Galle			X	Rate is worse; IMR is better
	Gampaha				Unable to determine
	Hambantota			X	Rate is worse; IMR is better
	Kaluthara	X			Rate and IMR are worse
	Kandy	X			Rate and IMR are worse
	Kegalle	X			Rate and IMR are worse
	Kurunegala		X		Rate and IMR are better
	Matale	X			Rate and IMR are worse
	Matara		X		Rate and IMR are better
	Moneragala			X	Rate is worse; IMR is better
	Nuwara Eliya			X	Rate is worse; IMR is better
	Ratnapura			X	Rate is worse; IMR is better

Source: Central Bank of Sri Lanka; Dept. of Census and Statistics; Ministry of Healthcare and Nutrition (multiple years)

**Table 5:** Actual and Counterfactual MMR at the District Level, Sri Lanka 1982–2002.

		Actual MMR per 1,000		Counterfactual Based on Pre-War Trend	Average Annual Change		Conflict Induced Outcome
Province	District	1982	2002	2002	Pre-conflict Average (1975 to 1982)	Average Change During Conflict	
High Conflict Level	Batticaloa	1.30	0.04*	0.50*	-0.054	-0.028*	Rate is worse; outcome is better
	Jaffna	0.10	0.40	0.01	-0.107	-0.012	Rate and outcome are worse
	Kilinochi	n/a	1.43*	n/a	n/a	n/a	Unable to determine
	Mannar	0.90	0.63*	0.45*	-0.014	0.069*	Rate is better; outcome is worse
	Mullaitivu	n/a	0.21*	n/a	n/a	n/a	Unable to determine
	Trincomalee	0.70	n/a	0.37	-0.032	-0.025*	Rate is worse
	Vavuniya	0.30	0.60*	0.06*	-0.107	-0.010	Rate is worse; outcome is same
Intermittent Conflict	Ampara	0.60	0.17	0.12	-0.077	-0.046	Rate and outcome are worse
	Anuradhapura	0.20	0.19	0.02	-0.119	-0.036	Rate and outcome are worse
	Polonnaruwa	0.40	0.14	0.07	-0.086	-0.047	Rate and outcome are worse
	Puttalam	0.30	0.21	0.03	-0.107	-0.043	Rate and outcome are worse
No Conflict	Badulla	0.80	0.28	0.27	-0.061	-0.023	Rate and outcome are worse
	Colombo	0.50	0.12	0.08	-0.088	-0.038	Rate and outcome are worse
	Galle	0.60	0.11	0.23	-0.047	-0.042	Rate is worse; outcome is better
	Gampaha	0.30	0.12	n/a	n/a	-0.022	Unable to determine
	Hambantota	0.80	0.15	0.05	-0.102	-0.033	Rate and outcome are worse
	Kandy	0.80	0.10	0.18	-0.071	-0.042	Rate is worse; outcome is better
	Kaluthara	0.50	0.06	0.11	-0.071	-0.042	Rate is worse; outcome is better
	Kegalle	0.60	0.12*	0.13*	-0.086	-0.040*	Rate is worse; outcome is better
	Kurunegala	0.60	0.20	0.23	-0.048	-0.035	Rate is worse; outcome is better
	Matale	1.00	0.12*	0.80*	-0.013	-0.043*	Rate and outcome are better
	Matara	0.90	0.14	0.53	-0.026	-0.043	Rate and outcome are better
	Moneragala	0.60	0.15	0.16	-0.065	-0.045	Rate is worse; outcome is better
	Nuwara Eliya	1.20	0.52	0.37	-0.057	-0.025	Rate and outcome are worse
	Ratnapura	0.70	0.31	0.11	-0.087	-0.035	Rate and outcome are worse

* Data from 1999 used as endpoint

Source: Central Bank of Sri Lanka; Dept. of Census and Statistics; Ministry of Healthcare and Nutrition (multiple years)

**Table 6:** Severity of Impact of Conflict on MMR at the District Level, Sri Lanka 1982–2002.

		Rate of change and MMR are worse than predicted	Rate of change and MMR are better than predicted	Rate of change is worse but MMR is better	Conflict Induced MMR
Province	District				
High Conflict Level	Batticaloa*			X	Rate is worse; MMR is better
	Jaffna	X			Rate and MMR are worse
	Kilinochi				Unable to determine
	Mannar*				Rate is better; MMR is worse
	Mullaitivu				Unable to determine
	Trincomalee*				Rate is worse
	Vavuniya*			X	Rate is worse; MMR is same
** **	** **				** **
Intermittent Conflict	Ampara	X			Rate and MMR are worse
	Anuradhapura	X			Rate and MMR are worse
	Polonnaruwa	X			Rate and MMR are worse
	Puttalam	X			Rate and MMR are worse
					
No Conflict	Badulla	X			Rate and MMR are worse
	Colombo	X			Rate and MMR are worse
	Galle			X	Rate is worse; MMR is better
	Gampaha				Unable to determine
	Hambantota	X			Rate and MMR are worse
	Kandy			X	Rate is worse; MMR is better
	Kaluthara			X	Rate is worse; MMR is better
	Kegalle*			X	Rate is worse; MMR is better
	Kurunegala			X	Rate is worse; MMR is better
	Matale*		X		Rate and MMR are better
	Matara		X		Rate and MMR are better
	Moneragala			X	Rate is worse; MMR is better
	Nuwara Eliya	X			Rate and MMR are worse
	Ratnapura	X			Rate and MMR are worse

* Data from 1999 used as endpoint

Source: Central Bank of Sri Lanka; Dept. of Census and Statistics; Ministry of Healthcare and Nutrition (multiple years)

The district-level analysis suggests that conflict exacted a toll on both IMR and MMR. Outcomes in the conflict zone are among the poorest experienced nationwide, but there is an interesting differential across IMR and MMR. Although poor, the IMRs do not prove to be the worst in conflict-districts. However, the conflict districts of Kilinochchi, Mannar and Vavuniya have the worst MMR outcomes. Overall health trajectories slowed in and around conflict zones, and throughout much of the country.

Results in districts within the Northern province were mixed. With only 17 recorded deaths per 1,000 live births, Jaffna district had one of the lowest IMRs in in the country prior to the start of the war [34–36]. By 2002, it obtained 6.0, a better outcome than the 12.7 per 1,000 yielded by the counterfactual model. Like Jaffna, Vavuniya achieved a comparatively low IMR of 16 per 1,000 in the pre-war years. By 2002, this was reduced to 11.8 per 1,000 –a poor performance when compared to the counterfactual outcome of 4.8. Mannar performed better than expected achieving a decrease in IMR from 27 per 1,000 to 6.9. The absolute gain and overall trend experienced during war were better than expected given the pre-war performance. Neither Kilinochchi nor Mullaitivu existed as independent districts prior to the war and so it is impossible to create a counterfactual. By 2002, the districts achieved an IMR of 3.9 and 8.9 respectively.

Three of the five districts in the Northern Province had the highest MMRs of all districts by 2002, despite the fact that the MMR per 1,000 live births in Jaffna and Vavuniya were among the lowest in the nation prior to the war. This changed dramatically over the course of two decades. By 1999, Kilinochchi’s rate of 1.43 per 1,000 live births was ten times higher than the national average of 0.14. Mannar’s 6.3 and Vavuniya’s 6.0 were also markedly higher than national average. Jaffna’s end rate of .39 per 1,000 was four times higher than the pre-war rate of 0.10 per 1,000. Likewise, Vavuniya’s pre-war rate of 0.3 was half the rate observed in 2002. Further, both Jaffna and Mannar’s 2002 MMRs were worse than predicted by the counterfactual model.

Development also stalled in the eastern districts of Ampara, Batticaloa, and Trincomalee. Ampara and Batticaloa failed to maintain prewar momentum in decreasing the IMR and ended up with IMRs that are higher than expected given the prewar trends. Batticaloa’s end rate of 15.2 is among the worst seen in the nation. Although Trincomalee averaged a decrease of 0.06 per 1,000 prior to the war, and this average decreased to only 0.043 during the war. Trincomalee ended up with a better than predicted IMR of 2.5. Ampara’s IMR of 7.0 was worse than predicted.

The pace of decline in maternal mortality slowed in all three districts. Despite the loss of momentum, Batticaloa ended up with an MMR of .038 per 1,000. This is a lower (better) rate than the 0.502 predicted by the counterfactual model. The MMR in Batticaloa is better than expected given the pre-war trend. Ampara has a slightly worse-than-expected outcome of 0.174 compared to the expected 0.121.

The two districts that constitute the North Central Province, Anuradhapura and Polonnaruwa, share the largest border with the conflict zone. Little changed in terms of population health during the conflict and both districts performed worse compared to what the counterfactual models suggest was possible. In fact, Anuradhapura and Polonnaruwa have the highest IMRs of all districts. Their IMRs (2002) are 17.6 and 16.1 per 1,000 live births respectively. By comparison, Batticaloa has an IMR of 15.2 and Vavuniya 11.8. Even though the IMR is high, Anuradhapura’s IMR did decrease from 26 to 17.6 over two decades; Polonnaruwa’s IMR actually got worse, increasing from 14 to 16.1. Neither district realized the potential inherent in pre-war momentum. The rate of change in IMR during the war years is slower than the pre-war momentum. The MMR in Anuradhapura shows virtually no improvement during the war, moving from 0.20 per 1,000 to 0.19 per 1,000 over two decades. The end rate is much higher than the expected rate of 0.016 per 1,000. Polonnaruwa fared little better. Its MMR improved from 0.40 in 1982 to 0.14 by 2002 and was higher than the predicted outcome of 0.07 per 1,000.

Badulla and Moneragala districts present a mixed story. Moneragala shares a more extensive border with the conflict region than does Badulla, but is farther south where fighting was arguably less intense. The rate of change for both IMR and MMR in Moneragala during hostilities was slower than the pre-war rate. Despite this, the final outcomes are better than predicted by the counterfactual model. In contrast, although Badulla’s IMR improved, the gains were not close to those one would expect given the pre-war pattern. The actual IMR of 15.9 per 1,000 is worse than the 10.7 predicted by the counterfactual model. The actual MMR is also marginally worse than expected.

Puttalam, Matale, and Hambantota also border, albeit in a small way, the conflict zone. These districts do not reflect any consistent outcome. The rate of change in the IMR during conflict was slower in Hambantota and Matale than was seen prior to 1983. The actual IMR outcome was worse than expected only in Matale. Puttalam performed better than expected and achieved an IMR of 6.3 per 1,000 in 2002. Both Puttalam and Hambantota did worse in MMR in both rate and absolute outcome as a result of the war. Puttalam’s MMR of .21 in 2002 is worse than the predicted MMR of .031. The story is similar for Hambantota. On the other hand, Matale’s outcomes were better than expected.

Outcomes in other districts suggest a modest impact of war on IMR and a more notable impact on MMR. Districts in the Central province experienced a decline in health. Both the rate of change and final IMR were worse than expected in two of the three districts. Furthermore, the rate of change for MMR slowed in two of three districts, with final outcome worse in Nuwara Eliya. Nuwara Eliya had one of the worst MMRs prior to the onset of war; by 2002 it had the second-worst MMR in the country and failed to achieve gains that would be expected given the pre-war rate of change at both district and national levels. Kurunegala’s overall performance in IMR is better than expected in both absolute outcome and rate of change. It did see a slowdown in change in MMR. Galle experienced a slowdown in decreasing the IMR, as did Ratnapura. Kegalle experienced worse IMR trends and absolute outcomes. The MMR in Ratnapura, and Colombo are higher than expected, while the rate of change is worse. Galle’s final MMR of 10.9 per 1,000 is better than expected despite a slowdown in the rate of change. Matara achieved an MMR of 5.9 per 1,000 which is also better than expected.

### Cause of Hospitalization

Sri Lanka’s pattern of hospitalization indicates that the nation is in the second stage of the epidemiological transition in that both chronic and communicable diseases account for the primary causes of hospitalization (Table 7). That traumatic injury is the primary cause of hospitalization nation-wide, and that it is the primary cause of hospitalization in the majority of districts is a morbidity pattern typical in nations moving from low to middle development [34–36]. However, the rise of traumatic injury may also be indicative of the social upheaval and violence of a war-torn country. In 1995, eleven districts recorded traumatic injury as the primary cause of hospitalization. By 2002, all but one district, Batticaloa, reported trauma as the primary cause. Nationally from 1980 to 2003 infectious disease was decreasingly important as a cause of hospitalization, moving from 2,054.2 cases per 100,000 in 1980 to 1,855.7 per 100,000 in 2003 [35]. Deaths from infectious disease likewise declined from 23.9 per 100,000 to 10.0 per 100,000 [35]. Concurrently, hospitalization due to injury, poisoning, and other external causes increased from 1731.8 per 100,000 in 1980 to 3,371.7 per 100,000 in 2003 [35]. Diseases of the circulatory system also rose during this time [35]. Hospitalization due to various neoplasms rose from 128.3 per 100,000 in 1980 to 276.2 per 100,000 in 2003[35]. Both epidemiological transition and better diagnostic availability may account for this trend.

**Table 7:** Top Five Leading Causes of Hospitalization by Province and District, 1995 and 2002.

		1995						2002				
Province	District	1	2	3	4	5		1	2	3	4	5
Sri Lanka		Traumatic injuries	Respiratory Disease	Symptoms, Signs	Intestinal Infectious disease	Viral disease		Traumatic injuries	Respiratory Disease	Viral disease	Symptoms, Signs	Gastrointestinal Tract disease
**Eastern**	**Ampara**	n/a						Traumatic injuries	Respiratory Tract	Viral diseases	Intestinal infectious diseases	Gastrointestinal Tract disease
	**Batticaloa**	Respiratory Disease	Traumatic injuries	Gastrointestinal Tract disease	Intestinal Infection	Musculoskeletal system disease		Respiratory Disease	Intestinal Tract	Traumatic injuries	Malaria	Intestinal infectious diseases
	**Trincomalee**	Traumatic injuries	Respiratory Disease	Malaria	Symptoms, Signs	Skin and subcutaneous tissue disease		Traumatic injuries	Respiratory Tract	Symptoms, signs	Intestinal Tract	Viral diseases
** **	** **											
**Northern**	**Jaffna**	n/a						Traumatic injuries	Respiratory Tract	Symptoms, signs	Musculoskeletal system and connective tissues (M00—M99)	Gastrointestinal Tract disease
	**Kilinochchi**	n/a						n/a	n/a	n/a	n/a	n/a
	**Mannar**	Respiratory Disease	Traumatic injuries	Malaria	Symptoms, Signs	Viral diseases		Traumatic injuries	Respiratory Tract	Intestinal infectious diseases	Malaria	Gastrointestinal Tract disease
	**Vavuniya**	Respiratory Disease	Traumatic injuries	Malaria	Symptoms, Signs	Viral diseases		Traumatic injuries	Symptoms, Signs	Respiratory Disease	Eye and Adnexa	Gastrointestinal Tract disease
	**Mullaitivu**	n/a						Traumatic injuries	Symptoms, signs	Respiratory System	Malaria	Skin and subcutaneous tissue

Source: Ministry of Healthcare and Nutrition (multiple years)

At the district level, there are several items of note. Table 7 shows that malaria plays a major role in hospitalization in the conflict districts of Vavuniya, Mullaitivu and Batticaloa. No other districts recorded malaria in the top five causes of hospitalization [35–37]. Jaffna, also a conflict district, reports malaria in its top 20 causes of hospitalization [36]. Although Sri Lanka has long struggled to control malaria, the continued impact of this disease in these areas is striking. Work in conflict zones has shown that morbidity and mortality from infectious disease increases [2, 6, 8, 20].

Neither national nor district level data differentiate type of traumatic injury. The data aggregate ICD codes 800–904, 930–939, 950–957 (1995 data ICD 9 codes) and ICD 10 codes S00-T19 (2002). Much like the nation as a whole, traumatic injuries account for the top cause of hospitalization in six of the seven conflict districts. It may be reasonable to suspect that trauma recorded in Trincomalee, Vavuniya and Mannar has a very different manifestation than that experienced across Sri Lanka as a whole, although one cannot make such a determination from the data at hand.

### Cause of Mortality

Only minor changes occurred in the primary causes of hospital-based death at the national level. There was no change in the top five causes of death (Table 8) and only small shifts among the top ten causes [36, 38]. It is interesting to note that perinatal mortality decreased in prominence over time. Perinatal mortality was important in hospital deaths for many years prior to 1995 [39, 40]. However, the burden from perinatal disease decreased by 2000 [37]. By 2002, direct and indirect obstetric causes remained an important cause for hospitalization (in the top 20), but deaths associated with the perinatal period were no longer listed in the top ten causes of hospital-based deaths [36]. That death associated with the perinatal period was no longer a top cause of hospital-based death could indicate that women began receiving better access to care during their pregnancies, and deliveries were being made in more sanitary settings. Thus, the national trend is for a decline in perinatal mortality. This makes the districts in which maternal and/or infant mortality increase stand out even more.

**Table 8:** Top Five Leading Causes of Hospital-based Mortality by Province and District, 1995 and 2002.

		1995					2002				
Province	District	1	2	3	4	5	1	2	3	4	5
Sri Lanka		Ischaemic Heart Disease	Cerebro-vascular Disease	Gastro-intestinal Tract	Pulmonary Circulation	Neoplasms	Ischaemic Heart Disease	Gastro-intestinal Tract	Pulmonary Circulation	Cerebro-vascular Disease	Neoplasms
**Eastern**	Ampara	n/a	n/a	n/a	n/a	n/a	Ischaemic Heart Disease	Pulmonary Circulation	Respiratory System	Cerebro-vascular Disease	Slow fetal growth, fetal malnutrition
	Batticaloa	Pulmonary Circulation	Poisoning (non-pesticide)	Gastro-intestinal Tract*	Intestinal Infectious Disease*	Ischemic Heart Disease	Slow fetal growth, fetal mal-nutrition	Respiratory Tract	Gastro-intestinal Tract	Neoplasms	Obstetric Conditions
	Trincomalee	Ischaemic Heart Disease	Other Bacterial Disease	Respiratory System	Cerebrovascular Disease	Pesticide Poisoning	Ischaemic Heart Disease	Gastro-intestinal Tract	Pulmonary Circulation	Symptoms, Signs	Respiratory System
** **											
**Northern**	Jaffna	n/a	n/a	n/a	n/a	n/a	Pulmonary Circulation	Zoonotic and other Bacterial Diseases	Ischaemic Heart Disease	Respiratory System	Cerebro-vascular Disease
	Kilinochchi	n/a	n/a	n/a	n/a	n/a	n/a	n/a	n/a	n/a	n/a
	Mannar	Ischaemic Heart Disease	Pneumonia	Symptoms, Signs	Ishaemic Heart Disease	Respiratory System	Ischaemic Heart Disease	Pulmonary Circulation	Zoonotic and other Bacterial Diseases*	Viral Disease*	Pesticide Poisoning
	Vavuniya	Listed with Manner					Pulmonary Circulation	Cerebro-vascular Disease**	Urinary System**	Ischaemic Heart Disease	Slow fetal growth, fetal malnutrition
	Mullaitivu	n/a	n/a	n/a	n/a	n/a	Malaria	Slow fetal growth, fetal malnutrition	Viral Disease	Zoonotic and other Bacterial Infections***	Gastro-intestinal Tract***

Source: Ministry of Healthcare and Nutrition, various years

* Listed as third leading cause of hospital-based death

** Listed as second leading cause of hospital-based death

*** Listed as fourth leading cause of hospital-based death

District level data from the conflict areas reflect a different pattern of mortality than that of the nation as a whole. They tell of a health toll from perinatal problems, infectious disease, and neglect. Slow fetal growth and malnutrition was one of the five most important causes of death in Batticaloa, Vavuniya, Mullaitivu, and Ampara in 2002. The situation appeared particularly dire for pregnant women in Batticaloa. Not only was slow fetal growth and malnutrition the primary cause of mortality, obstetric causes were the fifth most important cause of death. A pregnant woman was nearly as likely to lose her life as she was to see her baby die (Table 8).

Communicable diseases were also rampant. Malaria was the primary killer in Mullaitivu. Viral disease, zoonotic disease and bacterial infections were in the top five causes of death for Vavuniya, Mannar, Jaffna and Mullaitivu. Zoonotic/bacterial diseases were in the top five causes of death in only one additional district outside of the conflict area, but were in the top ten causes of hospital-based mortality in four additional districts [36]. Still, the weight of burden from these districts placed zoonotic/bacterial infections in the top ten national killers.

The portrait of death changed between 1995 and 2002 in much of the conflict area. Vavuniya and Mannar saw communicable diseases and slow fetal growth/malnutrition become primary causes of death [36, 38]. Batticaloa saw maternal and perinatal health failures cause more deaths per capita than pulmonary and heart disease[36, 38].

### Index of Conflict Impact on Population Health

**Fig 2:**
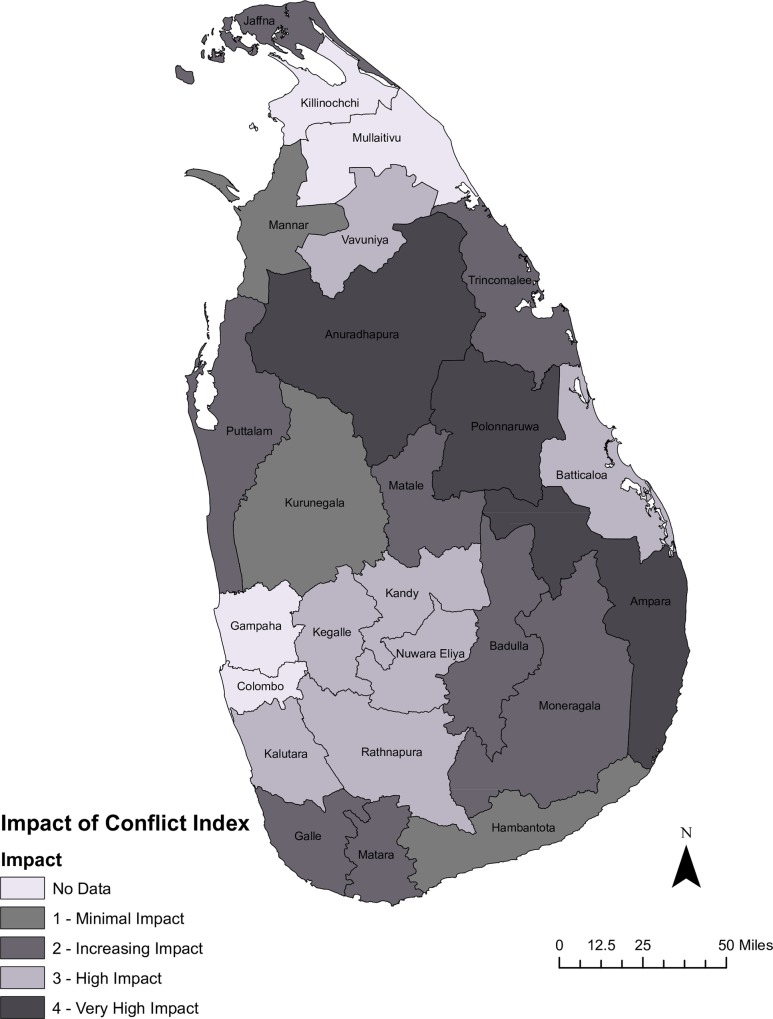
Index of Conflict Impact on Sri Lanka.

The majority of districts experienced a noticeable level of impact from conflict. Ten districts have a conflict weight of three or four, while an additional eight districts experienced poorer performance in half of the indicators than one would expect had conflict not occurred. Only three districts appeared relatively unscathed (Table 9). The lack of data for conflict districts of Kilinochchi, and Mullaitivu–areas within Vanni—is unfortunate but not unexpected. The districts which suffered the worst are those that border conflict area, but that the impact of conflict was far-reaching (Fig 2). The dry zone districts of Anuradhapura and Polonnaruwa, and the district of Ampara all show a conflict impact weight of four. The districts which demonstrated medium impact due to conflict include two districts within the conflict zone and five in the south central regions of the country. Moderate impacts are found within the conflict zone in Jaffna and Trincomalee, and districts away from the zone. There is no observable pattern to the three districts which experienced the lowest level of conflict impact in that Mannar, Kurunegala and Matara are scattered around the country.

**Table 9:** Impact of conflict on districts.

Weight	Interpretation	Number of Districts
N/A	Insufficient data for analysis	4
0	No loss in health	0
1	One indicator is worse than predicted; impact minimal	3
2	Two indicators worse than predicted; impact minimal	8
3	Three indicators worse than predicted; impact noticeable	7
4	All indicators worse than predicted; impact high	3

## Discussion

Sri Lanka is a study in the dynamics of long-term conflict, human health and development. Some 100,000 people are believed to have died during the course of the war, with anywhere from 9,000 to 40,000 killed in the brutal endgame [41]. These numbers belie the story that is told by national level data, based on which it would appear that despite a 26 year war, Sri Lanka experienced improvement in health and development. If true, this suggests that a) early achievements in human development shielded the country from some of the harsher impacts of protracted conflict; b) the effects of war were geographically isolated and did not spill over into broader society; c) the intensity of conflict varied across several decades and, as such, the peaks and valleys may have had negligible impact on long term processes that impact health and development; and/or d) protracted conflict interacted positively with development outcomes.

This analysis suggests that these ideas should be treated with caution. The invisible costs of war in Sri Lanka are not limited to excess mortality and morbidity during and after war, but include excess losses due to the diversion of resources away from the development path. Further, the impact was not geographically contained to conflict-intense districts. There are clear regional disparities with a noticeable decrease in health outcomes in conflict-intense districts in both actual and counterfactual outcomes. However, this analysis showed although conflict intense areas and proximate areas suffered from the war, negative health impacts were not limited to these areas. The level of development Sri Lanka had obtained prior to the war did not protect the country from begin thrown into a different, less-successful development path.

Losses in health were much more visible at the district than national level. Given previous work on disaggregation of health impacts, this outcome was not unexpected [2, 20]. Although nationally IMR and MMR improved during the course of the war, both could have perhaps improved even more. The rate of change in infant mortality decelerated during the war years and the actual IMR (2002) was poorer than could have been obtained had pre-war momentum continued. Based on pre-war trends, an additional 1 to 3 lives per 1,000 births were lost during the progress of the first two stages of the war. Maternal mortality proved to perform better than expected at the national level but many districts were left behind.

Sixteen districts experienced a slowdown in IMR improvement; of these, ten also had IMRs that were lower than would have would have been expected without a war. Three of the districts that experienced both a decline in rate of change and worse-than-expected outcomes were within the conflict zone and two bordered it. The remainder were not geographically proximate. Only five districts had better-than-expected outcomes. That infant mortality improved in Jaffna and Mannar was puzzling and will be discussed in further detail below.

The apparent conflict-effect is more pronounced in the maternal mortality ratios where we see that twenty districts were impacted. Of these, the rate of change decelerated in nine districts, in one the actual outcome was worse than expected, and a full ten districts both lost traction and had poorer-than-expected outcomes. Districts around the country suffered. Perinatal causes, malnutrition and infectious disease increased as causes of hospitalization and mortality during the of war in conflict areas. This suggests that public health measures which proved successful in the past were failing to adjust to the context of war and is consistent with other studies [2, 20, 42]. The health care system was proving unable to deliver care. Qualitative reports describe a lack of personal security. Such security is impacted through increased difficulties in accessing care, and in the destruction of health service centers and health service disruption [21, 42–44].

What do these outcomes, when taken together, tell us? The story told by IMR and MMR suggest that there are both direct costs, in the form of poorer-than-expected health outcomes, and opportunity costs stemming from loss of momentum in the health system. The impact index (Fig 2) shows that the three most heavily impacted districts, which had an impact factor of 4, were not in the central war zone but were proximate to it. Five of the second hardest hit districts did not even border the conflict zone. This challenges previous work that suggests the health impact of war is most concentrated in conflict-intense regions, but which failed to account for pre-war conditions [22, 45].The impact of conflict is most evident in Ampara, Anuradhapura, and Polonnaruwa, followed by Jaffna, Trincomalee, Puttalam, Matale, Badulla and Moneragala, Hambantota and Galle. Although Ampara was not the focal point of the war, it experienced frequent violence due in part to its proximity to the conflict zone. It is also the only district in the country in which the majority ethnic group is Muslim rather than Sinhalese or Tamil. The Muslims suffered violence from both of the combatant groups [28, 46, 47]. The poor outcomes may reflect not only the direct violence from the war but structural violence. Deterioration of physical infrastructure and systematic discrimination against the Muslims may have inhibited not only quality of but access to it as well. Compounding these factors may be cultural practices within the communities that heighten vulnerability to perinatal health problems, one of the main causes of hospitalization and death [42]. Although most deliveries in Sri Lanka occur in facilities, concern for personal security and religious beliefs may have inhibited facility-based delivery, as did poor road conditions.

Jaffna went from having the lowest MMR to having one of the higher rates over the course of war. Jaffna’s MMR escalated from 0.1 per 1,000 live births in 1982 (in a consistent range for five years prior to the war) to 0.7 in the mid-1980s. The rate began decreasing and then stabilized through most of late 1990s, reaching 0.4 (0.399) in 2002. What (re)development occurred after 1995 may have helped to lower the MMR from the higher rates of the late 1980s and early 1990s, but was not able to return the rate to it previous low. The 2002 rate was the poorest in the nation. Further, the 0.4 is higher than the 0.01 rate projected by the counter-factual model. Research conducted in 2004 found that the Northern Province (consisting of the districts of Jaffna, Kilinochchi, Mullaitivu, Vavuniy and Mannar) suffered highest rates of maternal mortality compared to other regions and in country as well as a dearth of health personnel, and access to safe water and sanitation [21].

Trincomallee lost momentum during the course of the war. Its pre-war rate of 0.7 was in the mid-range of the country. The 1990 outcome of 0.12 was better than that predicted by the model, but still, the rate of change was worse.

What was surprising was the apparent success in improving IMR in Jaffna and Mannar, and the apparent success in protecting Mannar from decline. These outcomes resonate with the theory of system complexity and suggest that multiple factors must be considered [48]. Within such a theoretical framing, an inflow of aid from international actors, increased coordination among actors, and increased health sector spending may all contribute to the counter-intuitive outcome of improved health in conflict zones [17, 22]. Jaffna was an administrative and trading hub, and the focus of colonial and national investment for decades (1930s forward) prior to the war. This may account for Jaffna’s comparatively high health achievements up to 1982. The LTTE controlled Jaffna from 1986 to 1995. When the GoSL recaptured Jaffna in 1995, it invested heavily in rebuilding infrastructure, livelihoods and public programs. The low IMR recorded in 2002 may reflect an aggressive program of rebuilding health, and through health, government legitimacy in the district. The IMR reported for Jaffna in 1996, 1997 and 1998 was 17.5, 11.4, and 10.4 respectively. It dropped dramatically to 5.9 in 1999; whether this is a reflection of true progress, more accurate reporting, or data manipulation is impossible to say.

Mannar experienced improvements in both IMR and MMR. In 1982, the IMR was 27 and had been relatively stable for five years. It took extreme swings during the course of the conflict, reaching a record high of 41.7 in 1991, dropping to 5.3 the following year, then going back up 17.8 in 1998. The rate was 6.9 in 2002. The swings may be due to data issues as centralized health data collection is inconsistent in warzones [33]. Work in Nepal suggested that health outcomes may continue to improve in conflict zones so long as there is no intentional disruption of health services. Throughout this time, the GoSL maintained health personnel and facilities in the region [37–40]. The MMR fell from 0.9 in 1982 to 0.63 in 2002. There was considerable static across the years. The rate was at 1.1 in 1996, rose to 1.9 in 1997 and was at 063 in 1999 [37–40].

The poorer than expected performance in the remaining districts adds support to the idea that the indirect impact of conflict is as great if not greater than the direct costs as none of these districts were the epicenter of the war [3, 5–8].

Anuradhapura and Polonnaruwa are historically poor districts. Both are situated in the Dry Zone and have small economies dominated by agriculture. Both districts are also home to ancient ruins, designated part of the Sri Lankan Cultural Triangle, that are UNESCO World Heritage sites. This area experienced spillover from the conflict zone. Transport and communication systems through much of this region were destroyed, as were irrigation systems and livestock vital to agriculture, which is the main economic driver of the area [49]. This further suggests an indirect impact from conflict in that the economy may have been disrupted. This, in turn, may have a negative multiplier effect on health and human development. The location of the Cultural Triangle suggests that there is potential to develop the area for tourism. However, the risk associated with the war and the general destruction in the area discouraged such investment through much of the conflict period [49, 50]. Private investment in this, and other regions, decreased during the war [49]. The result is that these districts suffered opportunity costs from the war.

Nuwara Eliya is historically underdeveloped [30, 51, 52]. It continues to not be a priority for national investment in health. Nuwara Eliya consistently had the worst IMR, and one of the worst MMRs in the country prior to the war. In 1982, the IMR was 49 per 1,000 and MMR was 1.2. Although both improved during the war, the actual rate of change and outcome of the MMR, and rate of change in IMR is poorer than expected given pre-war momentum. Its economy is dominated by agriculture. It is also a district dominated by the so-called Indian or Plantation Tamils, a group of ethnic Tamils who historically arrived in Sri Lanka starting in the nineteenth century. This is a collective which, although ethnically Tamil, has a political and social identity distinct from the Tamil population in the northern areas. The infant mortality rate among the Indian Tamils is nearly double that of the nation as a whole [30] and may be confounding the data. There is a long history of poor health and poor health investment in the district. Prior to national independence, the worst health outcomes in Ceylon were in urban areas and in the Tamil plantation area [30]. From 1940 to 1948, Nuwara Eliya benefitted from government investment in health and health protection. As a result, health status improved [30]. However, following the disenfranchisement of the Indian Tamils in 1948, these Tamils lost voting representation and lost government healthcare. The plantations themselves were to provide for the health of their workers. The result is that, yet again, health deteriorated and health in this area lagged behind that of the rest of the country [30]. In the 1980s, the Indian Tamils had voting rights restored and in the 1990s the plantation healthcare was nationalized. However, the potential to have renewed investment in health vis-à-vis political representation never became manifest. Whether it was due to the draw the war placed on resources and political will, or ineffective political power is difficult to determine. The war certainly did not help make health or development for this group anymore of a priority for government investment.

**Fig 3:**
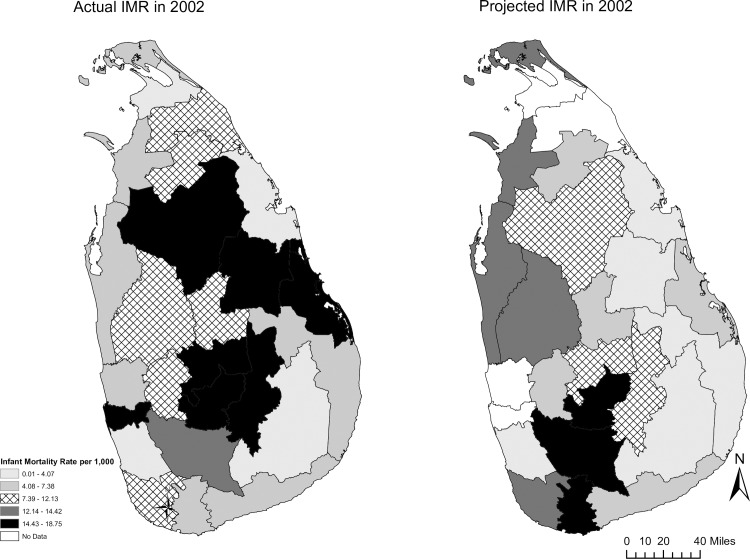
Actual and Projected Infant Mortality Rate in 2002. Source: Central Bank of Sri Lanka; Dept. of Census and Statistics; Ministry of Healthcare and Nutrition (multiple years).

**Fig 4:**
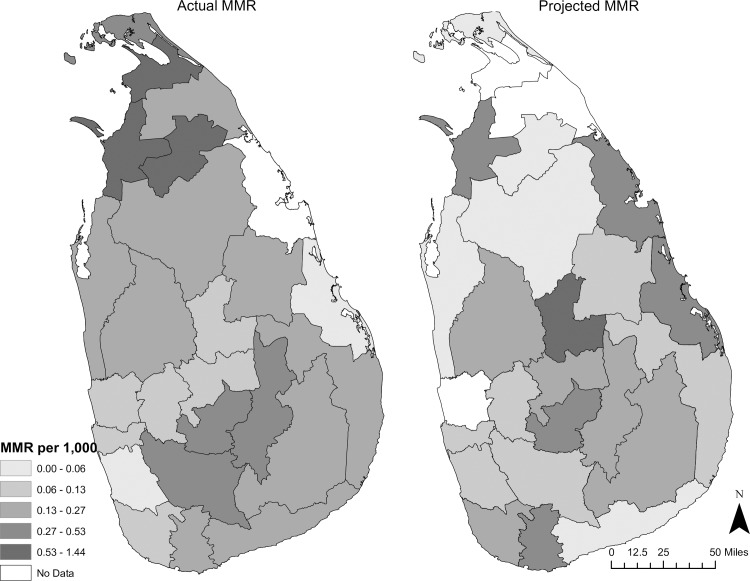
Actual and Projected Maternal Mortality Ratio in 2002. Source: Central Bank of Sri Lanka; Dept. of Census and Statistics; Ministry of Healthcare and Nutrition (multiple years).

Given that we are looking at 30 years of data, given that the intensity of conflict varied over time, and given Sri Lanka’s achievements in public health and development prior to the war, we would expect to see on-going improvement in health. Nationally and within some districts, indicators were comparatively good. However, the magnitude of improvement fell short. Furthermore, there was a pronounced loss of momentum and poor outcomes in multiple regions. Snapshots of theactual IMR and MMR are compared to the IMR and MMR that Sri Lanka could potentially have achieved are below (Figs 3 and 4).

These losses are likely related to both direct and indirect impacts of war. The direct losses come in overall loss of life, and in the high presence of traumatic injuries. Indirect effects are seen in the changes in epidemiological patterns that showed poor performance in maternal and child health in both mortality rates and morbidity, in lost economic opportunities, in fractured livelihoods. Such losses come about through a decline in both availability and quality of inputs necessary to health–health care, income, shelter, food, a safe environment, and livelihoods. Conflict is estimated to have reduced Sri Lanka’s growth rate by 2–3% per year, thereby placing Sri Lanka at a much lower economic outcome than what was feasible given the nation’s pre-war capabilities [53]. The World Bank estimates that the average cost of a civil war is greater than 30 years of GDP growth [3]. This means that resources invested in health decreased. Livelihoods, small-scale economic projects, employment and household capacity were disrupted by war while lines of inequity and vulnerability were exacerbated. Access to appropriate health services decreased through loss of facilities and personnel, and through less direct means. Poor road conditions, difficulties navigating security check-points, fear of violence, and lack of transportation options may have prevented women from accessing appropriate delivery facilities and contributed to poor maternal and child health outcomes. The continued presence of infectious disease as a cause of both hospitalization and death speak to a decrease in public health–environmental interventions, prevention programs and health care facilities.

### Limitations

There are a number of limitations to this study. The war in Sri Lanka continued until 2009, whereas this analysis used 2002, the third national ceasefire, as its endpoint. This model therefore does not capture the entire dynamic of the conflict period. Health outcomes may have continued to deteriorate across the nation as a result. It is also feasible that, in light of the ceasefire and the rebuilding and development which followed the tsunami, there could have been improvements in health and development. District boundaries for two areas in highly contested Vanni changed during the course of the war making it impossible to compare data from the region in a meaningful way. The use of administrative boundaries as geographic catchment areas for the data analysis creates issues of in terms of aggregation. The average experience of health within the district-level measure may have little relationship to town, household or individual level experience. Further, it was not possible to ascertain if mortality/morbidity was recorded based on where a person received treatment or where the person resided. It is quite possible that those living near the invisible boundaries of the district crossed that very boundary for healthcare and thus, that health outcome was prescribed to the treatment district rather than the resident district. This could explain the pronounced health impact observed in districts bordering conflict areas. An avenue for future research could therefore seek to use community- or household-level surveys prior to and after a conflict to eliminate error these errors of aggregation.

The model itself had a number of limitations. It assumed consistent linear change in each of the health outcomes, but the assumption on linear progression may be faulty. I provided a sensitivity analysis to highlight demonstrate where different assumptions may lead to different conclusions (see S2 Table: Appendix 2). The MMR outcomes proved to be not sensitive to the ‘shape’ of progression in that outcomes remained consistent across linear, exponential and log linear models. Results for IMR did change according to the assumed ‘shape’ of the change. That MMR was consistent across model adds credence to the results of this analysis. It is important to note that MMR proved to be more greatly disrupted by conflict than did IMR.

The counter-factual model did not allow for endogenous or exogenous forces to otherwise affect changes in health outcomes. It further assumes the presence of conflict within a district creates an equal impact across district, province and nation. This research provides evidence that there are indirect costs of war, but did not demonstrate the causal pathway between conflict and poorer-than-expected outcomes. Given the complexity of the social and environmental factors which impact health outcomes, a more elaborate model is warranted. Multi-variate regression analysis may be a starting point for better understanding which interaction in direct and indirect routes to poor health. The greatest limitation is the counter-modeling itself as the model attempts to show what *could* have happened if history had taken a different course. There is no true way to validate the ‘what if’ scenario.

### Conclusion

Understanding the short- and long-term effects, the direct and indirect channels through which conflict affects health and human development is a complex task. The complexity stems in part from difficulties in capturing the call and response of variables in a multifaceted system, and from our inability to gather appropriate measures of these variables. I applied a novel methodology to better understand the direct and the opportunity costs of war on public health.

This research revealed a counter-narrative to the story of Sri Lanka, human development and war. In that narrative, health did not continue to improve and the development was not unaffected by the war. In fact, the disaggregated and counter-factual health outcomes were important signs of the overall decline in public health and human development across the nation. Perhaps more insidious was the lost momentum, and the loss of what could have been achieved in terms of infant and maternal deaths prevented, in terms of increased longevity, and in terms of the wealth and happiness that could have come with better health, in the absence of war.

## Supporting Information

S1 Table Appendix 1. (PDF)

S2 Table Appendix 2. (PDF)

S1 Dataset Master health database. (XLSX)
