# Supplementary material for: The Cost of War on Public Health: An Exploratory Method for Understanding the Impact of Conflict on Public Health in Sri Lanka
Source: PLoS One. 2017 Jan 12;12(1):e0166674. doi: 10.1371/journal.pone.0166674 (PMC5231380; doi:10.1371/journal.pone.0166674)
Supplement: S1 Table — (PDF) [file pone.0166674.s001.pdf]

Appendix 1

Change in development indicators in country with/without conflict
Comparision of Costa Rica, Malaysia and Sri Lanka 1982 - 2002

Indicator	Costa Rica	Malaysia	Sri Lanka Actual	Sri Lanka Fictive
**IMR 1982**	19.4	23.5	34	
**Change in IMR 1983 - 2002**	-8.8	-14.9	-15.8	
**Average Annual Change**	-0.0234	-0.0311	-0.0221	-0.02
**GDP per capita 1982 ($US Constant 2000)**	2733.29	2057.94	461.37	
**% change in GDP/Cap 1983 - 2002**	0.0243	0.0451	0.0416	
**Maternal Mortality Ratio per**	33 (2004)	(30) 2003	43 (2003)	
**Population 2002**	4,083,197	24,515,323	19,134,096	

Source: World Bank 2013
