# Supplementary material for: The Cost of War on Public Health: An Exploratory Method for Understanding the Impact of Conflict on Public Health in Sri Lanka
Source: PLoS One. 2017 Jan 12;12(1):e0166674. doi: 10.1371/journal.pone.0166674 (PMC5231380; doi:10.1371/journal.pone.0166674)
Supplement: S2 Table — (PDF) [file pone.0166674.s002.pdf]

Appendix 2 Sensitivity analysis for district level outcomes

MMR Sensitivity Analysis

The table depicts: "The actual outcome is XXXX compared to the projected outcome"

	NATIONAL		CENTRAL			EASTERN			NORTH CENTRAL		NORTH WESTERN		NORTHERN					Sabaragamuwa		SOUTHERN			UVA		WESTERN		
	5 yr trend	10 yr trend	Kandy	Matale	Nuwara Eliya	Batticaloa (1999)	Ampara	Trincomalee (1999)	Anuradhapura	Polonnaruwa	Kurunegala	Puttalam	Jaffna	Kilinochchi	Mannar (1999)	Vavuniya (1996)	Mullaitivu	Ratnapura	Kegalle	Galle	Matara	Hambanto	Badulla	Moneraga	Colombo	Gampaha	Kaluthara
Linear	Worse	Better	Better	Better	Worse	Better	Worse	Better	Worse	Worse	Better	Worse	Worse	n/a	Worse	Better	n/a	Worse	Better	Better	Better	Better	Worse	Better	Worse	n/a	Better
Exponential	Worse	Worse	Better	Better	Worse	Better	Worse	Better	Worse	Worse	Worse	Worse	Worse		Better	Worse		Worse	Worse	Worse	Better	Better	Worse	Better	Worse		Better
Log	Worse	Better	Better	Better	Worse	Better	Better	Better	Worse	Worse	Better	Worse	Worse		Better	Better		Worse	Worse	Better	Better	Better	Better	Better	Worse		Better
**OUTCOMES**	C	I	C	C	C	C	I	C	C	C	I	C	C		I	I		C	I	I	C	C	I	C	C		C

IMR Sensitivity Analysis

This table depicts: "The actual outcome is XXX compared to the projected outcome"

	NATIONAL		CENTRAL			EASTERN			NORTH CENTRAL		NORTH WESTERN		NORTHERN					Sabaragamuwa		SOUTHERN			UVA		WESTERN		
	5 yr trend	10 yr trend	Kandy	Matale	Nuwara Eliya	Batticaloa	Ampara	Trincomalee	Anuradhapura	Polonnaruwa	Kurunegala	Puttalam	Jaffna	Kilinochchi	Mannar	Vauniya (1996)	Mullaitivu	Ratnapura	Kegalle	Galle	Matara	Hambanto	Badulla	Moneraga	Colombo	Gampaha	Kaluthara
Linear	Worse	Better	Worse	Worse	Better	Worse	Worse	Better	Worse	Worse	Better	Better	Better	n/a	Better	n/a	n/a	Better	Worse	Better	Better	Worse	Worse	Worse	n/a	n/a	Better
Exponential	Better	Better	Worse	Worse	Worse	Worse	Worse	Better	Worse	Worse	Worse	Better	Better	n/a	Better	n/a	n/a	Worse	Worse	Better	Better	Worse	Worse	Better	n/a	n/a	Worse
Log	Better	Worse	Better	Better	Better	Worse	Worse	Better	Worse	Worse	Better	Better	Better	n/a	Better	n/a	n/a	Better	Better	Better	Better	Better	Better	Worse	n/a	n/a	Better
**OUTCOMES**	I	I	I	I	I	C	C	C	C	C	I	C	C	n/a	C			I	I	C	C	I	I	I			I

C = Consistent

I = Inconsistent One of the two alternatives matched the linear model

D = disagreement; neither alternative matched linear model

C = Consistent
